# Silencing of Amyloid Precursor Protein Expression Using a New Engineered Delta Ribozyme

**DOI:** 10.1155/2012/947147

**Published:** 2012-02-12

**Authors:** Manel Ben Aissa, Marie-Claude April, Lucien-Junior Bergeron, Jean-Pierre Perreault, Georges Levesque

**Affiliations:** ^1^Département de Psychiatrie-Neurosciences, Faculté de Médecine, Unviersité Laval et Neurosciences CHUL, 2705 Laurier, Québec, QC, Canada G1V 4G2; ^2^RNA Group/Groupe ARN, Département de Biochimie, Faculté de Médecine et des Sciences de la Santé, Université de Sherbrooke, 3001 12th Avenue, Sherbrooke, QC, Canada J1H 5N4

## Abstract

Alzheimer's disease (AD) etiological studies suggest that an elevation in amyloid-*β* peptides (A*β*) level contributes to aggregations of the peptide and subsequent development of the disease. The major constituent of these amyloid peptides is the 1 to 40–42 residue peptide (A*β*_40−42_) derived from amyloid protein precursor (APP). Most likely, reducing A*β* levels in the brain may block both its aggregation and neurotoxicity and would be beneficial for patients with AD. Among the several possible ways to lower A*β* accumulation in the cells, we have selectively chosen to target the primary step in the A*β* cascade, namely, to reduce APP gene expression. Toward this end, we engineered specific SOFA-HDV ribozymes, a new generation of catalytic RNA tools, to decrease APP mRNA levels. Additionally, we demonstrated that APP-ribozymes are effective at decreasing APP mRNA and protein levels as well as A*β* levels in neuronal cells. Our results could lay the groundwork for a new protective treatment for AD.

## 1. Introduction

Alzheimer's disease (AD) is a degenerative disorder of the human central nervous system (CNS). Its clinical and neuropathological features are defined by a progressive loss of cognitive function and by the onset of a slowly progressive impairment of memory during mid- to late-adult life. The neuropathological hallmarks of AD include the accumulation and aggregation of amyloid-*β* peptide (A*β*), neurofibrillary tangles, astrocytic gliosis, and reductions in the numbers of both neurons and synapses in many areas of the brain, particularly in the cerebral cortex and hippocampus [1]. Strong evidence from multiple studies suggests that defects in A*β* regulation are one of the central biochemical events leading to the development of AD [2]. The neurotoxic A*β* fragment originates from the amyloid protein precursor (APP) following sequential cleavages by *β* (BACE) and *γ*-secretases (presenilin complex). Observations on the physiological processing of APP and on the effects of pathogenic mutations in the APP and/or the presenilin genes have led to the hypothesis that aberrant processing of APP into A*β* peptides is linked to AD [3]. We have previously reported strong evidence indicating that the amyloid cascade is an early and critical event in the neurodegeneration associated with AD. For example, cell lines and/or transgenic mice expressing mutant presenilin 1 (PS1), presenilin 2 (PS2), or APP exhibit an accelerated rate of neurotoxic A*β* formation [4]. Thus, the three known genetic causes of familial AD affect A*β* metabolism. Moreover, the *ε*4 allele of apolipoprotein E, a strong genetic risk factor for the development of AD, has been linked to either enhancing A*β* aggregation or decreasing its clearance in brain tissue [5, 6]. Altogether, these observations strongly suggest that targeting A*β* metabolism is a *worthwhile therapeutic approach* and that reducing its level in the brain may block both the neurodegenerative process and cognitive decline. Most likely, an approach that reduces either the level of A*β* or the rate of its aggregation and deposition in the brain would be beneficial for patients with AD. Targeting the secretases may be risky because they appear to have multiple roles in cells. We have decided to address the problem with a new generation of ribozymes (Rz) targeting the first step in the amyloid cascade, specifically, the APP mRNA.

**Figure 1:**
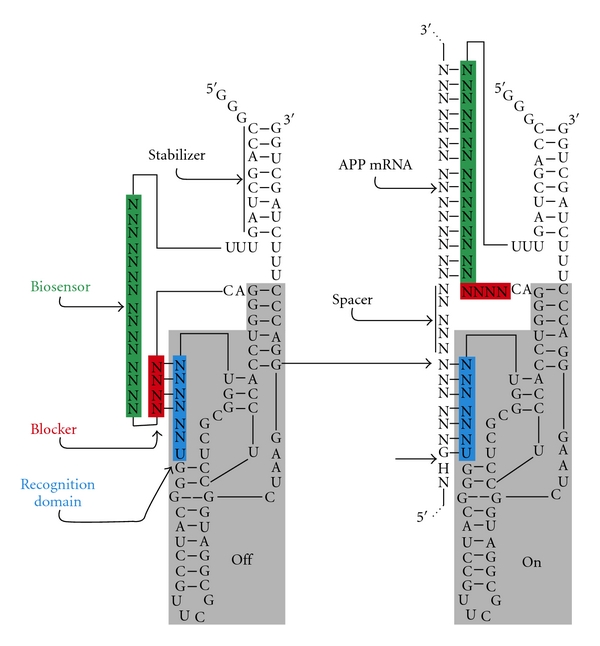
Secondary structure of both the *Off* and *On* conformations of the SOFA-HDV ribozyme. The original HDV ribozymes in the grey shaded boxes with its recognition domain (in blue) indicated. The SOFA is composed of three motifs : the biosensor (in green), blocker (in red), and stabilizer stem. Upon the addition of APP mRNA, sequence-specific hybridization to the ribozyme occurs, and the substrate is subsequently cleaved.

Hepatitis Delta Virus ribozyme (HDV Rz) is an interesting potential tool for the development of a gene-inactivation system because it is well adapted to the human cell environment [7]. In fact, this ribozyme offers several unique properties, including the natural ability to function in the presence of human proteins and at physiological magnesium concentrations as well as outstanding molecular stability (i.e., it has a long half-life) [8]. Recently, a novel target-dependent ribozyme that increases HDV Rz fidelity was engineered [9]. This new ribozyme possesses a module (the SOFA, for Specific On/Off Adaptor) that switches the cleavage activity from *Off* to *On* when in the presence of the appropriate substrate (Figure 1). Specifically, this module is composed of three domains: a blocker, a biosensor, and a stabilizer. The blocker sequence inhibits the cleavage activity of the ribozyme by intramolecularly binding the recognition domain of the Rz, which was limited to only 7 nucleotides before the addition of the module. Binding of the blocker switches the ribozyme domain to an inactive state, namely, the *Off* conformation. Upon addition of the substrate, the biosensor binds its complementary sequence on the substrate and unlocks the SOFA module, thereby permitting a switch of the ribozyme into the active fold, namely, the *On* conformation. The sequences of the substrate binding of both the ribozyme recognition and biosensor domains are not contiguous, but rather are separated by a small region called the spacer that varies from 4 to 7 nucleotides for optimal design [10]. Finally, the presence of a stem (namely, a stabilizer) that brings together both the 5′ and 3′ extremities has no effect on the cleavage activity but stabilizes the SOFA-HDV Rz *in vivo* against ribonucleases. A proof of concept of this man-made ribozyme has been demonstrated both *in vitro* and *in vivo* using ribozymes that cleaved various mRNA and viral RNA [11–13]. The fact that the SOFA-HDV Rz is activated by its mRNA substrate greatly diminishes its nonspecific effects; consequently, it displays significant potential for applications in both functional genomics and gene therapy.

In this study, we evaluated the potential of the new SOFA-HDV ribozymes as an RNA silencing tool in mammalian cells. In cell culture, we demonstrated the effects of SOFA-HDV Rz targeting APP mRNA on A*β* production.

## 2. Experimental Procedures

### 2.1. SOFA-HDV Ribozyme DNA Constructs

SOFA-HDV ribozymes were constructed using a PCR-based strategy that included two complementary and overlapping oligonucleotides. Briefly, two DNA oligonucleotides were synthesized and annealed with the reverse primer (5′-CCAGCTAGAAAGGGTCCCTTAGCCATCCGCGAACGGATGCCCA(N)_6(P1)_ACCGCGAGGAGGTGGACCCTG(N)_4(BL)_) and the sense primer (5′-TTAATACGACTCACTATAGGGCCAGCTAGTTT(N)_12(BS)_(N)_4(BL)_CAGGGTCCACC), where N is A, C, G, or T, and P1, BS, and BL indicate the P1, biosensor, and blocker sequences, respectively. It is important to note that both the P1 and BS segments were varied to correspond to specific APP mRNA sequences and that the BL was complementary to the first 4 nucleotides on the 5′ end of the Rz's recognition domain. For *in vitro* synthesis of the ribozymes, the sense primer also included the sequence of the T7 RNA polymerase promoter at the 5′ end. The filling reaction was performed in a 100-*μ*L volume containing 20 mM Tris-HCl (pH 8.8), 10 mM KCl, 10 mM (NH_4_)_2_SO_4_, 2 mM MgSO_4_, 0.1% Triton X-100, 2 *μ*M of each dNTP, 1 *μ*M of each DNA oligo, and 5 U of Pwo DNA polymerase (Roche Diagnostics). The reactions were ethanol precipitated and washed and the DNA pellets resuspended in 56 *μ*L of deionized water. The resulting PCR products were directly used for *in vitro* transcription reactions (see below). For the *in cellulo* experiments, the PCR products were inserted into the *Eco*RV site of pCDNA3 (Invitrogen). The SOFA-HDV-Rz cassettes were removed by digestion with *Bam*HI and subcloned into pRNAT-U6.1/lentivector (GenScript) under control of the U6 snRNA promoter. The resulting plasmids were named pRNAT-SOFA-HDV-Rz-APPX, where X represents the APP cleavage position.

### 2.2. *In Vitro* Transcription of SOFA-HDV Rz and APP mRNA

RNA transcriptions were performed as previously described [14]. In the case of the SOFA-HDV ribozymes, the resuspended DNA pellets were used in 100-*μ*L transcription reactions containing 80 mM HEPES-KOH (pH 7.5), 24 mM MgCl_2_, 2 mM spermidine, 40 mM DTT, 5 mM of each rNTP, 0.01 U of pyrophosphatase (Roche Diagnostics), 24 U of RNAGuard (Amersham Biosciences), and 10 *μ*g of purified T7 RNA polymerase and allowed to proceed for 4 h at 37°C. The reactions were then treated with 4 U of RQ1 DNase (Promega), phenol-chloroform extracted, ethanol precipitated, and washed. Following these steps, the RNA pellets were resuspended in 40 *μ*L of deionized water. One volume of loading buffer (97.5% formamide, 0.05% bromophenol blue, 0.05% xylene cyanol, 10 mM EDTA) was added, and the samples were fractionated by 8% denaturing (8 M urea) polyacrylamide gel electrophoresis (PAGE, 19 : 1 ratio of acrylamide to bisacrylamide), using 45 mM Tris-borate (pH 7.5) and 1 mM EDTA. The RNA bands were visualized by UV shadowing, and the gel slices were cut out and eluted overnight using 500 mM ammonium acetate, 1 mM EDTA, and 0.1% SDS. After ethanol precipitation, the RNA transcripts were resuspended in deionized water and quantified by UV absorbance at 260 nm. The plasmid pAPP12 (containing a full-length copy of the APP mRNA) was used as a template. After linearization by the *Stu*I restriction enzyme, mRNA was synthesized as described above and purified using 5% PAGE. After purification, the transcripts (40 pmol) were dephosphorylated in a final volume of 50 *μ*L containing 200 mM Tris-HCl (pH 8.0), 10 U RNAGuard, and 0.2 U of calf intestinal alkaline phosphatase (Amersham BioSciences) at 37°C for 30 min. The reactions were purified by extracting twice with phenol : chloroform, and the mRNA was then precipitated with ethanol, washed with 70% ethanol, and dried. Dephosphorylated RNA (~6 pmol) was 5′-end-labeled in a final volume of 10 *μ*L containing 3.2 pmol of [*γ*-^32^P]ATP (6000 Ci/mmol, New England Nuclear), 10 mM Tris-HCl (pH 7.5), 10 mM MgCl_2_, 50 mM KCl, and 3 U of T4 polynucleotide kinase (United States Biochemicals) at 37°C for 90 min. The reaction was stopped by the addition of formamide dye buffer (5 *μ*L), and the reaction mixtures were fractionated through denaturing 5% PAGE gels and recovered as described above.

### 2.3. Ribonuclease H Probing and Primer Extension Assays

Ribonuclease H (RNase H) reactions were performed with a library of randomized oligonucleotides (5′-N_6_CD-3′, where N is for any A, C, G, or T residue and D is for any A, T, or G residue). Specifically, nonradioactive APP mRNA (0.5 *μ*M) and randomized oligonucleotides (5 *μ*M) were preincubated for 10 min at 25°C in a final volume of 8 *μ*L containing 20 mM Tris-HCl (pH 7.5), 20 mM KCl, 10 mM MgCl_2_, 0.1 mM EDTA, and 0.1 mM DTT. RNase H (0.5 U, United States Biochemicals) was then added, and the samples were incubated at 37°C for 30 min. After the incubation, 90 *μ*L of water was added, and the mixture was phenol : chloroform extracted. The nucleic acids were then precipitated with ethanol, washed, and dried. Four DNA oligonucleotides complementary to the APP RNA were purchased from Invitrogen (5′-GTTCCTCAGCCTCTTCCT-3′ (position 928-911), 5′-TCAGCCAGTGGGCAACAC-3′ (position 719-702), 5′-GTCAGGAACGAGAAGGGC-3′ (position 540-523), and 5′-CTGAATCCCACTTCCCAT-3′ (position 310-293)). The oligonucleotides (10 pmol) were 5′-end-labeled as described above. The ^32^P-end-labeled oligonucleotides were purified with denaturing 20% PAGE, and the relevant bands were excised from the gel and eluted overnight at 25°C, passed through a G-25 spun column, ethanol precipitated, washed, dried, and dissolved in deionized water (60 *μ*L). 5′-^32^P-labeled primer (6 *μ*L) and 10X reverse transcriptase buffer (0.6 *μ*L of 500 mM Tris-HCl (pH 8.3), 800 mM KCl, and 100 mM MgCl_2_) were used to resuspend the pellets resulting from the RNase H hydrolysis. The primer annealing step was performed by successively incubating the samples at 65°C for 2 min followed by 2 min on ice. The reactions were initiated by adding 0.8 mM of each dNTP, 3.3 mM DTT, and 100 U of Superscript II Reverse transcriptase (Invitrogen) in a final volume of 12 *μ*L. The samples were incubated at 45°C for 30 min and then ethanol precipitated and analyzed by 5% sequencing PAGE. DNA sequencing reactions using the same primer were migrated on the same gels to allow for identification of the primer extension stops. The results were visualized with a PhosphorImager.

### 2.4. Ribozyme Cleavage *In Vitro*

Cleavage reactions were carried out under single turnover conditions ([Rz] ≫ [S]), as previously described [15]. Specifically, ^32^P-end-labeled APP mRNA (50 nM) was mixed with SOFA-HDV ribozymes (1 *μ*M) in a 10-*μ*L mixture containing 50 mM Tris-HCl (pH 7.5) and 10 mM MgCl_2_ and then incubated at 37°C for 1 h. The reactions were stopped by the addition of loading buffer, RNA fractionated with denaturing 5% PAGE, and analyzed with a PhosphorImager.

### 2.5. Cell Culture and Transfection

A subclone of the human embryonic kidney cell line HEK-293 (tsA-201 cells, which were kindly provided by Dr. Mohamed Chahine, Laval University) and human neuroblastoma SH-SY5Y cells (ATCC) were cultured in Dulbecco's modified Eagle's medium (DMEM, Gibco) supplemented with 10% (v/v) fetal bovine serum (Biomedia). Stock cultures were maintained at 37°C in a humidified atmosphere with 5% CO_2_. The HEK-293 cells were transiently transfected with pRNAT-SOFA-HDV-Rz-APPX (Rz-APP-X) plasmid using the calcium phosphate procedure. The empty pRNAT-U6.1 vector (GenScript) was used as a control. The SH-SY5Y cells were transduced using a lentiviral system. This system consists of the multiply deleted packaging construct pCMVΔR8.91 (which encodes Gag, Pol, Tat, and Rev), the pMD.G expressing vesicular stomatitis virus (VSV-G) surface glycoprotein (G), and pRNAT-U6 (either with or without Rz-APPX). To produce the infectious virions, HEK-293 cells (2 × 10^6^) were plated on 5 dishes (10 cm) and transfected the next day with 20 *μ*g of Rz-APPX, 15 *μ*g pCMVΔR8.91, and 5 *μ*g pMDG using the calcium phosphate procedure. Conditioned medium was harvested at 48 hr after transfection, cleared of debris by low-speed centrifugation, and filtered through 0.45 *μ*m filters (Sarstedt). The filtrate containing the virions was concentrated by ultracentrifugation at 71,000 × g for 90 minutes at 16°C using a SW-40 Beckman rotor, followed by a second cycle of centrifugation for the collected and resuspended pellets at 84,000 × g for 90 min (using a 4-mL centrifuge tube; SW60 Beckman rotor). Virions pellets were then resuspended in 0.5 mL of phosphate buffered saline (PBS). SHSY-5Y cells were infected with 0.2 mL of the virions expressing SOFA-HDV-Rz-APPX in the presence of 6 *μ*g/mL polybrene (hexadimethrine bromide, Sigma). Three days postinfection, the medium was replaced with medium containing 600 *μ*g/mL G418 for selection. The transduced cells were maintained as a stable population. The culture medium was changed every 3-4 days for the duration of the experiment.

### 2.6. SOFA-HDV Ribozyme Expression

To test the expression of the Rz-APPX, total RNA was extracted from transduced cells using the TRIzol reagent according to the manufacturer's recommendations (Invitrogen). Total RNA extracts were then used in primer extension experiments for ribozyme detection. Briefly, the primers, corresponding to the 3′ complementary sequence of either SOFA-HDV-RzX (5′-GGGTCCCTTAGCCATGCGCGAACG-3′) or U6 RNA (5′-GGCCATGCTAATCTTCTCTG-3′), were 5′-end-labeled with [*γ*-^32^P]ATP (6000 Ci/mmol; New England Nuclear), as previously published [13], annealed to 10 *μ*g of total RNA by a 5 min incubation at 65°C and immediately chilled on ice. The reactions were initiated with the addition of 0.4 mM of dNTPs, 10 mM DTT, and 200 units of Superscript II reverse transcriptase (Invitrogen) in a buffer containing 50 mM Tris-HCl (pH 8.3), 75 mM KCl, and 3 mM MgCl_2_ in a final volume of 50 *μ*L. The samples were incubated at 42°C for 50 min; the reactions were stopped by heating the samples to 70°C for 15 min and then fractionated through 10% denaturing polyacrylamide gel electrophoresis.

### 2.7. Real-Time RT-PCR

First-strand cDNA synthesis was performed using 2 *μ*g of total RNA in the presence of poly dT primers and 200 units of SuperScript II reverse transcriptase. Aliquots of 2 *μ*L from the resulting single-stranded cDNA products were used along with the appropriate primers (see below) for APP and GAPDH. Amplifications were performed for each sample from each separate well in a total volume of 25 *μ*L containing 1X SYBR Green Universal PCR Master Mix and 400 nM of specific forward and reverse primers. The primers were designed to overlap the boundaries of two exons (to avoid amplification of genomic DNA), using the Primer Express software v2.0 (Applied Biosystems). Specifically, two pairs of primers were designed to amplify the APP mRNA. The first pair (sense primer 5′-GGCGGTGTTGTCATAGCGA-3′ and antisense primer 5′-TGCATCTTGGACAGGTGGC-3′) provided an amplicon of 136 base pairs (bp), whereas the second pair (sense primers 5′-AACGAAGTTGAGCCTGTTGATG-3′ and antisense primer 5′-AACGAAGGCTGGCACAAC-3′) amplified a 67-bp fragment. Amplification of GAPDH mRNA using the sense primer 5′-CGACACTTCCAGCTCTTTGCT-3′ and antisense primer 5′-GAATCAGGGTTATCTGGTCATCG-3′, which produces an amplicon of 131 bp, was also performed. The PCR amplifications were performed on an ABI Prism 7000 Sequence Detector System (Applied Biosystems), according to the manufacturer's instructions and using the following conditions: 1 cycle at 95°C for 10 min, followed by 40 cycles at 95°C for 15 s, 58°C for 10 s, 72°C for 20 s, and a final step at 60°C for 60 s. The control samples were amplified without the reverse transcription step.

### 2.8. Preparation of Cell Lysates

Native and transfected cells were rinsed twice with ice-cold PBS and then lysed for 30 min on ice in cell lysis buffer containing 50 mM Tris (pH 7.6), 150 mM NaCl, 2 mM EDTA, 1% NP-40, 20 mM PMSF, and minicomplete protease inhibitors (Roche-Diagnostic). Insoluble material was removed by centrifugation at 13,000 × g for 15 min at 4°C. Finally, the proteins were quantified using a standard Bradford assay (Bio-Rad).

### 2.9. Western Blot Analysis of APP Processing

Western blot analysis was performed as previously described [16]. Briefly, 20 *μ*g of total protein from each sample was mixed with Novex 2X reducing sample buffer containing 500 mM Tris-HCl (pH 6.8), 20% glycerol, 10% SDS, 0.1% bromophenol blue, and 5% *β*-mercaptoethanol. The samples were then boiled for 5 min and subjected to SDS-PAGE. Following the migration, proteins were transferred onto a PVDF membrane (Millipore) according to the manufacturer's protocol. The membranes were probed with an anti-APP C-terminal antibody (A8717, Sigma-Aldrich) and *β*-tubulin specific antibody (antibody E7 for *β*-tubulin, Developmental Studies Hybridoma Bank). The blots were revealed using a chemiluminescence detection system (Immobilon Western, Millipore) according to the manufacturer's recommendations. The intensity of the signals was analyzed using image densitometry software (Imaging Densitometry, Bio-Rad). The level of *β*-tubulin was used to normalize the levels of APP (i.e., the ratio of APP versus *β*-tubulin) to control for differences in the loading of total proteins. Modulations of the APP levels in the cells treated with Rz-APP-X were expressed as a percentage of the level in the control cells (empty vector).

### 2.10. Quantitation of A*β* Using the Sandwich ELISA Method

Following SH-SY5Y transduction and during the selection, media was collected, preserved, and frozen at −80°C. Following, secreted A*β* was measured by sandwich ELISA, according the manufacturer's protocol (Human Amyloid *β* (A*β* 1-x) Assay Kit, IBL).

### 2.11. Statistical Analysis

For the *in vitro* data, the results from several experiments were analyzed using Student's *t*-test. Differences were considered significant at *P* < 0.05.

## 3. Results

### 3.1. Design and Selection of APP-Specific SOFA-HDV Ribozymes

**Figure 2:**
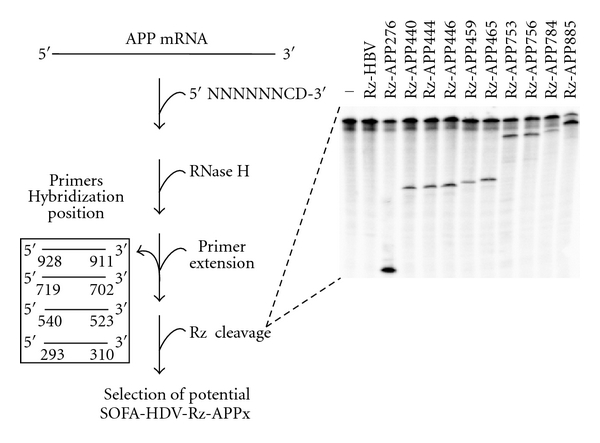
Selection of APP-SOFA-HDV ribozyme with the greatest potential cleavage. The left portion illustrates the strategy that was used to identify potential cleavage sites. APP mRNA was preincubated in the presence of a 7 nt long randomized DNA oligonucleotide, and RNA/DNA heteroduplexes were hydrolyzed by RNase H. Accessible regions were then visualized by primer extension using one of the four 5′-end-labeled primers (in box) complementary to sequences retrieved in the first *∼*900 nucleotides of the APP mRNA. Once the most accessible sites were identified, the appropriate SOFA-HDV ribozymes were synthesized and the cleavage activity was tested *in vitro* using 5′-end-labeled APP mRNA. A typical autoradiogram of a resulting PAGE is indicated in the right panel. The number of each Rz indicates the cleavage position within the APP mRNA. The Rz-HBV, previously used for HBV RNA cleavage [15], served as an irrelevant Rz. “-” indicates a reaction without Rz.

The first step of this study consisted of designing a collection of ribozymes that produced *in vitro* cleavage of the APP mRNA. Due to unfavorable competition with intramolecular base pairing, the target sequences located in single-stranded regions of an mRNA are potentially more accessible for Rz binding than those in double-stranded regions. It has been demonstrated that both target site accessibility and the ability to form an active ribozyme-substrate complex constitute interdependent factors that can be addressed using a combinatorial library of oligonucleotides or ribozymes [17]. To identify the cleavage sites with the greatest potential for targeting, we adopted a procedure based on the use of a library of partially randomized oligonucleotides mimicking the interaction with the recognition domain of the target [15] (Figure 2). In principle, all of the accessible sites within the APP mRNA should be specifically bound by an oligonucleotide and the resulting RNA-DNA heteroduplex subsequently hydrolyzed by RNase H. The resulting cleavage sites were identified by primer extension reactions using 5′-end-labeled primers, and the most potent SOFA-HDV ribozymes were tested for cleavage activity. The library was composed of oligonucleotides that were 8 nucleotides in length corresponding to one residue before the cleavage site (i.e., position -1), which had to be single stranded for cleavage to occur, and the 7 residues of the recognition domain of the ribozyme. It is important to note that this experiment considered only the binding domain of the ribozyme and not the SOFA module. It would be irrelevant to perform RNase H assays using long oligonucleotides that included the complementary sequence of the ribozyme's recognition domain, spacer and biosensor sequences. In that case, the spacer would also be bound, leading to significant formation of the duplexes and the introduction of an important bias. The library was designed while taking into consideration the sequence specificities of the HDV ribozyme. Specifically, the nucleotide in position 1 cannot be a guanosine; therefore, the 3′ end residue of the oligonucleotide cannot be a cytosine. Moreover, the first base between the ribozyme's recognition domain and the target must be a GU wobble base pair. Consequently, the oligonucleotide included a cytosine at the corresponding position. This constraint led to a library of 12,288 different variants corresponding to the 5′-N6CD-3′. The action of a ribozyme within the 5′-end of an mRNA region should enhance the probability that the cleavage product results in an RNA that cannot encode an active protein. Because each primer produced a readable sequence of 200 to 300 bases, 4 different oligonucleotides were designed for the reverse transcriptase reaction to analyze the first *∼*900 nucleotides of the APP transcripts corresponding to the 1040 nucleotides of the 5′ end (see Section 2). The relative level of accessibility in function of the intensity of the primer extension products is compiled in Table 1. This analysis led to the identification of 10 potential sites, located from positions 276 to 885, of the APP transcript. Seven of these sites appeared to be highly accessible, including 5 that were located near position 450. A high concentration of such sites in the same area is indicative of a single-stranded region, although it may also result from a synergetic effect of several oligonucleotides binding the same RNA transcript, resulting in unfolding of that region and increasing the possibility that additional oligonucleotides can also bind.

Subsequently, HDV-Rz with the appropriate recognition sequences was designed. To increase specificity, the ribozyme was further extended with the addition of a SOFA module. The resulting ribozymes were named SOFA-HDV-Rz-APPX, where X represents the APP cleavage position. The ability of these ribozymes to cleave the 5′-end-labeled APP transcripts was tested under single-turnover conditions ([Rz] ≫ [S]) and analyzed via PAGE (Figure 2 inset). Clearly, all of the SOFA-HDV ribozymes exhibited cleavage activity, although at different levels. Specifically, the cleavage level varied from 4% to 71%. Moreover, all of the ribozymes exhibited a specific cleavage at only the expected site. The 4 SOFA-HDV ribozymes that exhibited a cleavage level higher than 30% were conserved for the subsequent step. These ribozymes included SOFA-HDV-Rz-APP276, -APP753, -APP756, and -APP885, with cleavage activities of 62%, 36%, 36%, and 71%, respectively. These 4 potential SOFA-HDV ribozymes targeting the APP mRNA were tested with the ribosubstrates online software (http://www.riboclub.org/ribosubstrates). This integrated software searches selected cDNA databases for all of the potential substrates for a given SOFA-HDV ribozyme [18]. These potential substrates include not only mRNAs with perfect matches with the catalytic RNA tested, but also the wobble bp and mismatches. Interestingly, none of these 4 potential SOFA-HDV ribozymes seemed to have the potential for off-target effects (data not shown). Moreover, this analysis indicated that no other cleavage could occur within the APP gene family. Therefore, the chosen sequences were specific to APP mRNA. In other words, the SOFA-HDV ribozymes that exhibited significant cleavage activity *in vitro* against a derived APP transcript appeared to be specific to the APP mRNA.

### 3.2. Expression of APP-Specific SOFA-HDV Rz in Human Cells

In an attempt to achieve a high level of expression of SOFA-HDV ribozymes that maintain their affinity for the targeted mRNA, we adopted the pRNAT/U6 (which employs the U6 RNA polymerase III promoter) for a high level of small RNA expression. The advantage of this promoter is that RNA transcription terminates with the addition of 4 or 5 uridines (U) at the 3′-end, and this change has only a minimal effect on SOFA-HDV ribozyme folding based on RNA structure predictions. This approach also avoids nonspecific effects that might be caused by the transcription of additional regions of the vector sequence.

**Figure 3:**
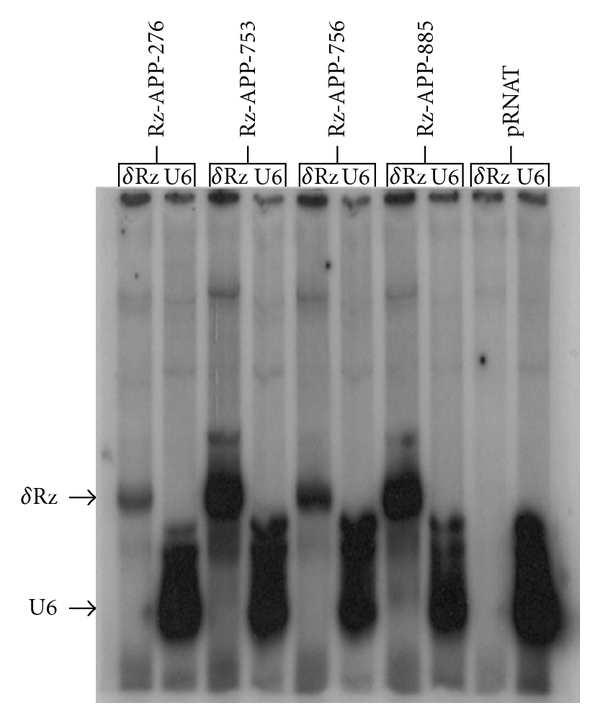
Expression of APP-SOFA-HDV ribozymes in the HEK cell line. Primer extension analysis of total cellular RNA from cells transfected with four selected APP-SOFA-HDV ribozymes from the ribozyme collection (APP-SOFA-HDV-Rz276, APP-SOFA-HDV-Rz753, APP-SOFA-HDV-Rz756, and APP-SOFA-HDV-Rz885). Transcripts corresponding to HDV-ribozymes were detected with HDV-Rz primers (5′-GGGTCCCTTAGCCATGCGCGAACG-3′). U6 primer (5′-GGCCATGCTAATCTTCTCTG-3′) was also used as a positive control, which yielded signals corresponding to endogenous U6 snRNA. Note that all of the 4 selected SOFA-HDV ribozymes were expressed (lanes 1 to 4). A pRNAT empty vector, was used as a negative control.

To determine whether the pRNAT/U6 SOFA-HDV-Rz-APPX vector could express the anti-APP SOFA-HDV ribozymes, these constructs were transfected into HEK-293 cells. Two days after transfection, total RNA from transfected cells was subjected to primer extension analysis. Endogenously synthesized U6 snRNA and SOFA-HDV ribozymes transcribed from the U6 promoter were detected, respectively, by U6- and Drz ^32^P-labeled primers (see Section 2). As indicated by Figure 3 (lanes 1 to 8), the specific extension products corresponding to U6 SOFA-HDV-Rz-APP276, -APP753, -APP756, and -APP885 were detectable in the transfected cells. An expression vector lacking a SOFA-HDV ribozyme coding sequence (pRNAT/U6) was used as a negative control. No detectable band of Drz ^32^P-labeled primers was observed with the empty vector (Figure 3, lane 9). The expression levels for SOFA-HDV-Rz-APP753 and -APP885 were among the highest, while two other ribozymes (SOFA-HDV-Rz-APP276, and -APP756) exhibited weaker expression.

### 3.3. Effect of Selected SOFA-HDV Ribozymes on APP mRNA Expression Level

**Figure 4:**
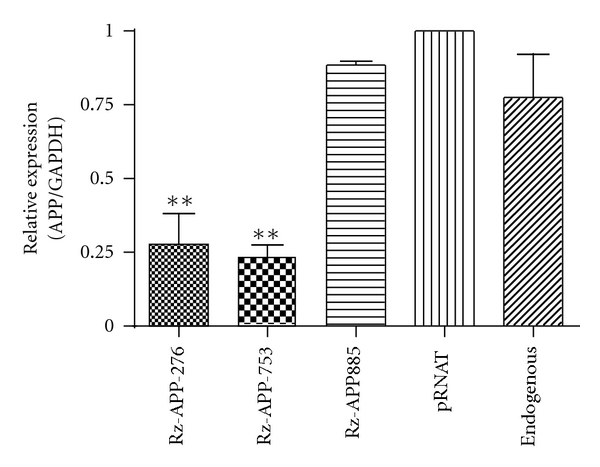
Relative APP mRNA level in HEK-293 cells expressing APP-SOFA-HDV Rz. The expression profile of APP in SOFA-HDV-Rz276-, -Rz753-, -Rz756-, and -Rz885-transfected HEK-293 cells. To assess APP knockdown efficiency at the mRNA level, quantitative real-time PCR was performed with each of the forward and reverse APP or GAPDH primers using the ABI Prism 7000 Sequence Detection System and SYBR Green DNA binding dye (Invitrogen). The specific amplification was assessed based on the dissociation curve profile. The APP gene expression profile was normalized against that of GAPDH. The quantitative PCR procedure was performed in duplicate in three independent reactions for each sample. **P < 0.01. An approximately 70–80% decrease in GAPDH-normalized APP mRNA levels was observed with cells expressing active SOFA-HDV Rz (the most potent Rz). The GAPDH-normalized levels of endogenous APP were not significantly altered in the untreated or empty vector-transfected control cells.

Considering the close and positive correlation between the level of APP mRNA, protein, and A*β* deposition in AD [19], APP mRNA expression levels following APP SOFA-HDV ribozyme expression were initially monitored. Previously, it has been shown that the SOFA-HDV Rz expressed in HEK-293 cells could be a powerful and specific gene silencing tool [9]. Therefore, SOFA-HDV-Rz-APP was transiently transfected into HEK-293 cells, which are well known for the expression of endogenous APP mRNA. pRNAT-U6 empty vector was used as a control. The total RNA was extracted from cells 48 h after transfection, and APP mRNA levels were quantified by real-time quantitative PCR (qPCR). The GAPDH mRNA was used as a control for the qPCR to normalize the APP mRNA levels. A significant effect of SOFA-HDV-Rz-APP ribozyme expression on cellular APP mRNA levels was observed (Figure 4). SOFA-HDV-Rz-APP276 and -APP753 transfection led to a highly significant (*P* < 0.001; Student's *t*-test) decrease in APP mRNA steady-state levels (*∼*70% and 80%, resp., relative to the control cells). Conversely, both SOFA-HDV-Rz-APP756 and -APP885 expression did not show a significant decrease at the APP mRNA level, suggesting that cleavage sites at positions 756 and 885 may not be as accessible *in cellulo *as they were in the *in vitro *assays on partial mRNA transcripts. The transfection of cells with an empty vector resulted in a faint increase in APP mRNA compared with untransfected cells (Figure 4, lanes 5 and 6), but this effect was not significant (*P* > 0.05; Student's *t*-test). More importantly, this experiment provided physical evidence that the expression of both SOFA-HDV-Rz-APP276 and -APP753 in HEK-293 cells resulted in an important decrease of the targeted APP mRNA levels and that nonspecific effects of vector transfection could not account for this decrease.

### 3.4. Effect of the SOFA-HDV Ribozyme on APP Protein Levels

**Figure 5:**
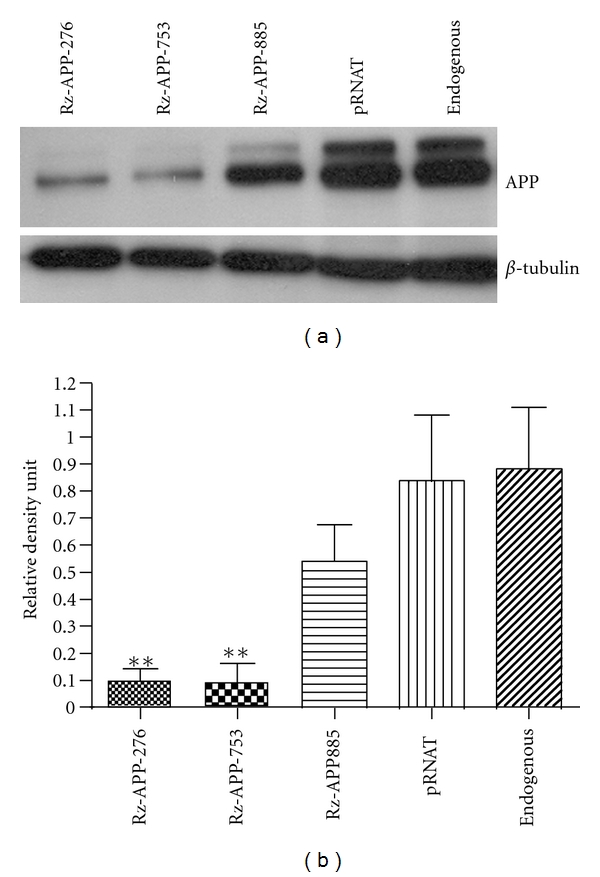
Rz-APP-X ribozymes expression reduces APP levels. (a) Western blotting analysis performed using equal amounts of 20 *μ*g of extracted protein. APP was immunodetected using a polyclonal antibody (Sigma) recognizing the C-terminus of human APP. The tubulin (*β*-tubulin) controlled the amount of sample loaded in each lane. The highest APP reduction (approximately 85%) was obtained with Rz-APP-276 and Rz-APP-753. (b) Densitometric quantification of the APP lanes in the blot from (a). Relative density unit values were obtained by standardization with the corresponding *β*-tubulin protein band in each lane. The results in (b) are presented as the means ± SEM from 3 independent experiments. **P < 0.01.

Because the correlation between the level of mRNA and its concomitant protein is not always linear, the effect of APP's directed SOFA-HDV ribozymes on APP protein levels was then investigated to verify whether the decrease in APP mRNA level results in a reduction at the protein level. SOFA-HDV-Rz-APP ribozymes were transfected into HEK-293 cells. As a control, cells were transfected with a pRNAT-U6 vector expressing GFP protein. At 48 h after transfection, the cells were lysed and total proteins were extracted. Subsequently, a Western blot was performed with a specific anti-APP C-terminal antibody as a probe. The level of APP was estimated by densitometry and normalized using endogenous *β*-tubulin (Figure 5). As expected, cells expressing both SOFA-HDV-Rz-APP276 and -APP753 showed a drastic decrease in APP levels compared with untransfected cells, those transfected with empty vector or those transfected with SOFA-HDV-Rz-APP885 (Figure 5(a)). The last construct exhibited a decrease estimated to be less than 20%, whereas the two other ribozymes led to reductions of over 70% relative to the controls. All of the changes were highly significant (*P* < 0.001) when compared with either untransfected cells or cells transfected with pRNAT/U6. Thus, the decrease in APP mRNA resulting from the expression of SOFA-HDV ribozyme is correlated with the change observed at the protein level. Moreover, these data are strongly consistent with the hypothesis that the expression level of APP mRNA is closely and positively correlated with its concomitant protein level [20].

### 3.5. Assessment of A*β* Secretion Levels in SOFA-HDV Ribozyme-Treated Cells

**Figure 6:**
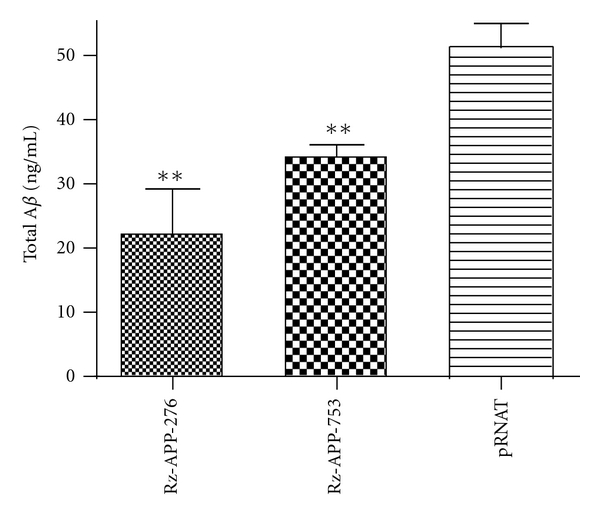
Effect of the most active ribozyme on the level of secreted total amyloid. The secreted levels of A*β*_1-x_ were detected by sandwich ELISA, as described in Section 2. Media were collected following SH-SY5Y infection with the Rz-APP-286, Rz-APP-753, or pRNAT empty vector. The results are presented as the means ± SEM from 3 independent experiments. **P < 0.001. Control cells were infected with the empty pRNAT vector.

From the perspective of AD therapy, any attempts to decrease APP mRNA levels should also result in a decrease in A*β* levels. To assess whether a decline of APP in ribozyme-treated cells leads to a decline in total A*β* levels, the level of secreted A*β* following SOFA-HDV-Rz-APP expression was determined by ELISA. For this experiment, the SOFA-HDV-Rz-APP276 and SOFA-HDV-Rz-APP753 were selected as the two more active and powerful ribozymes. Because neurons will be the target of the ribozymes in the context of AD, this ribozyme was tested on a neuronal cell type, SHSY-5Y, using a lentiviral system of expression. This system is essential for transducing neurons because post-mitotic cells cannot be efficiently transfected by other vectors. Following SHSY-5Y transduction, the SOFA-HDV-Rz-APP753 expression was tested for its effect on the reduction of APP at both the mRNA and protein levels (data not shown). To evaluate the A*β* level, media samples were collected and analyzed for A*β*_1-x_ species, as both A*β*_40_ and A*β*_42_ are associated with AD pathogenesis. Knocking down APP with lentiviral SOFA-HDV-RzAPP276 or OFA-HDV-RzAPP753 transduction of SHSY-5Y cells reduced the total level of A*β* by more than 30% (Figure 6). This result indicates that a SOFA-HDV ribozyme could be a potential means of targeting APP.

## 4. Discussion

In this study, we designed a new molecular tool to target the top of the amyloid cascade, namely, the APP mRNA. The SOFA-HDV ribozyme is based on a new synthetic HDV ribozyme harboring a biosensor module that activates mRNA cleavage only in the presence of the specific RNA target substrate [9]. This specific *On/Off* adapter (SOFA module) provides not only a higher specificity to the HDV Rz toward its target but also a higher cleavage capacity [10]. An initial experiment to identify the most susceptible site within the 5′ end region of the APP mRNA was performed based on the use of a randomized library of short oligonucleotides mimicking the recognition domain of the ribozyme. The hydrolysis of the formed RNA/DNA heteroduplexes by the RNase H led to the identification of 10 potential sites (Table 1). *In vitro* cleavage of a partial APP transcript by the corresponding appropriate SOFA-HDV ribozymes revealed that 4 of these sites could be cleaved at a significant level (Figure 2). Interestingly, an analysis of the sequence and secondary structure of the SOFA-HDV ribozymes that exhibited only moderate cleavage activity indicated that misfolding of 5 out of 6 of these ribozymes may explain their limited potential (data not shown). Therefore, only one of the SOFA-HDV ribozymes did not cleave efficiently for any specific reason. This result is excellent, considering that the initial analysis was based on the hybridization of small oligonucleotides and that the SOFA-HDV ribozyme is almost a magnitude larger in size but possesses a complex tertiary structure and two binding domains that interact with the substrate (i.e., the recognition and biosensor domains) and undergoes conformation transition [21]. 

The SOFA-HDV ribozymes exhibiting the highest cleavage activity *in vitro* were further studied *in cellulo*. These ribozymes were expressed from a U6 promoter for the RNA pol III because it allows efficient transcription of small RNA molecules [22]. According to the primer extension assays performed for these four SOFA-HDV ribozymes, they all exhibited good expression, although variable, in transfected HEK293 cells (Figure 3). Therefore, neither their ability to be expressed nor their stability accounted for their variable cleavage activity. Two of the SOFA-HDV ribozymes exhibited equivalent and drastic reductions in APP at both the mRNA and protein levels (Figures 4 and 5; SOFA-HDV Rz-APP276 and -APP753). It is not surprising that only some of the ribozymes that showed excellent cleavage activity *in vitro* exhibited significant cleavage activity *in cellulo*. Several different factors in cells compared with the *in vitro* analysis may account for this result. The *in cellulo* target is the full-length mRNA, which may adopt a different structure, and cellular proteins may be bound to it and create steric hindrances that reduce the accessibility to some of the identified cleavage sites.

To our knowledge, this study is the first reporting acute silencing of APP in human cells using an HDV ribozyme-based approach. SOFA-HDV ribozyme-based gene silencing constitutes an alternative to using small interfering RNA, a method that faces several limitations. One of the largest hurdles in RNAi-based therapy is toxicity. In this context, independent off-target or nonspecific effects of siRNA are a concern [23, 24]. Side effects can result from unintended interactions between an siRNA compound and an unrelated host gene. This nonspecific interaction with host genes may cause adverse effects in the host. Moreover, shRNA expression in neurons has been shown to interfere with dendritic spine structure and function, resulting in a decrease of synapses [25]. Interferon response is the best known adverse effect in the viral-mediated transfection of siRNAs. Similarly, synthetic siRNAs formulated in nonviral delivery vehicles can also be potent inducers of interferons and inflammatory cytokines, both *in vivo* in mice and *in vitro* in human blood [26]. The most important difference between ribozyme technology and RNAi technology is that RNAi requires the recruitment of endogenous proteins, which are responsible for the high intracellular activity. Thus, problems of potency, specificity, and/or cell-type-dependent responses illustrate a lack of understanding of the intracellular mechanisms involved [27]. By contrast, the HDV Rz, which derives from the hepatitis *delta *virus, possesses several unique features that are all related to the fact that it is the only naturally occurring catalytic RNA discovered in humans [7, 28, 29] and that its action does not depend on intracellular factors [30]. In addition, it exhibits an outstanding stability (i.e., a half-life > 100 hr) in cell culture [8]. Moreover, a genome-wide search for innate ribozyme entities revealed the presence of HDV-like sequences in the human CPEB3 gene [31]. Consequently, the HDV Rz should not be recognized by the immune system as an external, invading RNA.

Several studies in human genetic and animal models support the notion that amyloid production or accumulation in the brain plays a central role in the pathogenesis of AD. Lowering amyloid levels in different mouse models has demonstrated therapeutic value [32, 33]. Multiple approaches aimed at interfering with A*β* metabolism have been proposed as a therapy for AD. First, compounds that aim to decrease the aggregation of A*β* by blocking its oligomerization have been tested [34]. Although successful in mice models of AD, they failed in human trials. Second, lowering A*β* levels by increasing its clearance using a vaccine was successful in animal models [35, 36], but the results from human clinical studies indicated important side effects, and there were concerns about safety in humans [37]. Finally, blocking the activity of the secretases (*β* and *γ*) is attractive because both of these enzymes participate in A*β* production by cleaving APP. However, because these secretases have numerous substrates essential for cellular functions, blocking their activity raises additional concerns. The data from *β*-secretase (BACE1) knockout animals have suggested potential liabilities with BACE1 inhibition [38–40]. BACE1 is also known to participate in myelination [41–43] and the processing of sodium channels [43]. Nonetheless, BACE1 inhibitors have been developed, but blood-brain-barrier penetration and limited access to cellular BACE1 due to its major location in the endosome pose significant challenges that have yet to be overcome [44, 45]. Another potential therapeutic target is *γ*-secretase, although it has numerous essential cellular substrates. However, recent clinical trials testing a very promising *γ*-secretase inhibitor have raised major safety issues about this route [46, 47]. This failure does not question the amyloid hypothesis, but instead the nonspecific targeting of an enzyme complex with so many cellular functions [48].

Because there have been many failures in targeting amyloid peptide metabolism, we believe that targeting the top of the cascade by decreasing APP mRNA would be a better way to decrease the overall amyloid level. We do not expect a complete knock down of APP mRNA and A*β*, but we are confident that we can significantly reduce APP mRNA levels (and subsequently, A*β* levels). We believe that there is a threshold effect and that a modest reduction in A*β* levels could shift the balance between toxicity and nontoxicity. Another advantage of specifically targeting APP mRNA is that the level of all forms of the peptide derived from APP will also be decreased. To achieve this end, we engineered specific SOFA HDV ribozymes, a new generation of catalytic RNA tools, to decrease the APP mRNA level. We demonstrated that a SOFA-HDV ribozyme targeting APP mRNA is clearly effective for the reduction of A*β* in neuron-like cells. Further analysis using an expression system based on the lentivirus indicated a significant decrease of ~30% in total A*β* levels (Figure 6). Therefore, this action could possibly affect downstream amyloid-related pathology. Because only a 12% decrease in A*β* levels in mice resulted in a dramatic reduction in A*β* build-up and synaptic deficits [49], we are optimistic that the results obtained in our cellular model will allow for the development of an efficacious form of SOFA-HDV ribozyme-based therapy. The exact role of A*β* as a trigger of sporadic Alzheimer's disease is still a question of debate. Moreover it is actually not clear which A*β* species is associated with the disease. Over production of A*β* is probably not the cause of amyloid accumulation in sporadic AD. A defective clearance of amyloid may trigger its aggregation. This is supported by the fact that the ApoE4 allele, which is the major genetic risk factor for sporadic AD, slows down A*β* clearance [50]. Whatever the cause of A*β* accumulation, we believe that decreasing A*β* production by specifically targeting the APP mRNA will contribute to a decrease in the amyloid load to a non-toxic level. Actually, all therapies targeting A*β* (secretase inhibitors, vaccines, etc.) aim to reach this nontoxic level.

One limitation of our gene silencing approach is the delivery of the ribozyme to the nervous system. The presence of the blood-brain barrier limits the penetration of particle as large as a lentivirus into the central nervous system (CNS). To avoid this limitation local stereotaxic injections of lentiviruses could be used. Although this method is invasive, robust long-term and nontoxic lentiviral gene transfer is feasible in the rodent and nonhuman primate brains [51, 52]. Expression over 3 to 8 months can be achieved and it has been demonstrated that up to 90% of cells from the central nervous system transduced by a lentiviral vector under the control of the NSE promoter are neurons [53, 54]. However the method of choice for lentiviral delivery is the i.v. or i.p. route. This could be achieved by the fusion of the low-density lipoprotein receptor-binding domain of the apolipoprotein B to the therapeutic molecule. Successful application of this approach as a general method for the delivery of therapeutic molecules to the CNS has been demonstrated [55]. Moreover it remains possible that systemic presence of SOFA HDV ribozymes will decrease A*β* level in the periphery and concomitantly brain A*β* levels due to the “sink hypothesis.” Evaluation of these delivery methods will involve extending our study to animal models of Alzheimer's disease, thus the exciting potential of this new treatment will be revealed in the future.

This development will involve extending our study to animal models of Alzheimer's disease, so the exciting potential of this new treatment will be revealed in the future.

We have presented an original and unambiguous demonstration that a SOFA-HDV ribozyme can serve as an efficient gene silencing tool. Moreover, our results open the door to further evaluation of SOFA-HDV ribozymes as potential therapeutic molecules, or at least to a study demonstrating whether a reduction in A*β* levels is a viable therapy against Alzheimer's disease.

## Figures and Tables

**Table 1:** Determination of the most potential cleavage sites within APP mRNA.

Cleavage position	mRNA sequence^1^ recognition domain/biosensor domain	Accessibility^2^	SOFA-HDv Rz cleavage activity (%)^3^
276	**5′**-**G**CACAUG/CCAGAAUGGGAA-3′	++	62
440	**5′**-**G**CAAGCG/GCAAGCAGUGCA-3′	+++	18
444	**5′**-**G**CGGGGC/GCAGUGCAAGAC-3′	+++	20
446	**5′**-**G**GGGCCG/AGUGCAAGACCC-3′	+++	26
459	**5′**-**G**UGCAAG/UCCCCACUUUGU-3′	+++	14
465	**5′**-**G**ACCCAU/CUUUGUGAUUCC-3′	++	26
753	**5′**-**G**GAGGAU/GGAUGUCUGGUG-3′	++	36
756	**5′-G**GAUGAC/UGUCUGGUGGGG-3′	+	36
784	**5′-G**CAGACA/UAUGCAGAUGGG-3′	+	25
885	**5′-G**GACGAU/UGGUGAUGAGGU-3′	+	71

^1^ The sequences of the mRNA bound by both the ribozyme' recognition and biosensor domains. ^2^ Accessibility of potential cleavage sites based on RNase H hydrolysis. ^3^ Percentage of cleavage activity of the various SOFA-HDV ribozymes targeting the APP mRNA.
